# Functional Role of p53 in the Regulation of Chemical-Induced Oxidative Stress

**DOI:** 10.1155/2020/6039769

**Published:** 2020-02-28

**Authors:** Xiaoyi Liu, Lihong Fan, Chengrong Lu, Shutao Yin, Hongbo Hu

**Affiliations:** ^1^Beijing Advanced Innovation Center for Food Nutrition and Human Health, Department of Nutrition and Health, College of Food Science and Nutritional Engineering, China Agricultural University, 17 Qinghua East Road, Haidian District, Beijing 100083, China; ^2^College of Veterinary Medicine, China Agricultural University, 2 Yunamingyuan West Road, Haidian District, Beijing 100193, China; ^3^Air Force Medical Center of PLA, 30 Fucheng Road, Haidian District, Beijing 100142, China

## Abstract

The nuclear transcription factor p53, discovered in 1979, has a broad range of biological functions, primarily the regulation of apoptosis, the cell cycle, and DNA repair. In addition to these canonical functions, a growing body of evidence suggests that p53 plays an important role in regulating intracellular redox homeostasis through transcriptional and nontranscriptional mechanisms. Oxidative stress induction and p53 activation are common responses to chemical exposure and are suggested to play critical roles in chemical-induced toxicity. The activation of p53 can exert either prooxidant or antioxidant activity, depending on the context. In this review, we discuss the functional role of p53 in regulating chemical-induced oxidative stress, summarize the potential signaling pathways involved in p53's regulation of chemically mediated oxidative stress, and propose issues that should be addressed in future studies to improve understanding of the relationship between p53 and chemical-induced oxidative stress.

## 1. Introduction

An imbalance in the oxidation reduction (redox) system in favor of oxidants is known to cause oxidative stress, a condition that is characterized by the overproduction of reactive oxygen species (ROS) and/or decreased antioxidative capacity [1, 2]. Typical ROS include superoxide anion ^·^O_2_^−^, peroxide ^·^O_2_^−2^, hydrogen peroxide H_2_O_2_, hydroxyl radical ^·^OH, and hydroxyl OH^−^ ions. A number of cellular systems have been identified to contribute to ROS generation, including plasma membrane, cytosol, peroxisomes, mitochondria, and endoplasmic reticulum. Mechanistically, ROS generation is mainly due to excessive stimulation of NAD(P)H oxidases or the oxidative energy metabolism in mitochondria [3].

Oxidative stress has been shown to contribute to many pathological conditions, such as cancer [4–6], cardiovascular disease [7, 8], diabetes [9], neurodegenerative diseases [10, 11], and certain chemical-induced toxicities (Huo et al. 2016), [12–14]. Redox homeostasis is controlled by a battery of enzymes and nonenzymatic compounds [15, 16]. The oxidative stress-related enzymes include superoxide dismutases (SODs) [17], catalase [18], glutathione peroxidase (GPx) [19], heme oxygenase-1 (HO-1) [20], thioredoxins (TRXs) [21], peroxiredoxins (PRXs) [22], glutaredoxins [23], cytochromes P450 (CYPs), and nicotinamide adenine dinucleotide phosphate (NADPH) oxidase [7, 24]. Nonenzymatic redox-related molecules include mainly glutathione (GSH), ascorbic acid, and tocopherols/tocotrienols [25–27].

The major transcriptional factors involved in redox regulation include Nrf2, Nrf1, p53, and FoxO [14, 28–30]. Of these, p53 was the first to be identified and is the best known tumor suppressor. The primary functions of p53 include the regulation of cell cycle and apoptosis and the promotion of DNA repair [31]. In addition to these canonical activities, there is increasing evidence to suggest that p53 contributes to a number of noncanonical functions, such as the regulation of redox balance, glucose metabolism, and autophagy [32–34]. Moreover, p53 plays dual roles in the control of oxidative stress, as it can both exert prooxidant activity to promote oxidative damage and also function as an antioxidant factor to inhibit oxidative stress (as shown in Tables 1 and 2). These contradictory functions of p53 in the regulation of redox status may be associated with the particular conditions of the cells, which may be either stressed or nonstressed. Elucidating the complexities of p53 in the regulation of the redox balance will improve our understanding of the mechanisms that underlie the oxidative stress-mediated pathological conditions, which, in turn, will assist in the management of these redox imbalance-related diseases. This review focuses on the role of p53 in the regulation of chemical-induced oxidative stress.

## 2. Prooxidant Activity of p53 in Chemical-Induced Oxidative Stress

Oxidative stress can be activated in response to certain chemicals and has been demonstrated to play a critical role in their toxicity [12, 14, 35–38]. p53 is a sensor of cellular stress, and the induction of oxidative stress is, thus, generally accompanied by the activation of p53. In certain contexts, p53 activation functions as a prooxidant factor to promote oxidative stress-mediated toxicities. One example of such an environment is cancer chemotherapy, in which the side effects on normal tissues are common drawbacks, posing a major limitation on the efficacy of the treatment. The generation of ROS and activation of the p53 signaling pathway have been implicated in the side effects of a number of chemotherapeutic drugs [14, 35, 39].

Cisplatin is a first-line therapeutic drug for certain types of cancer; however, its nephrotoxicity is a major impediment to its clinical application [35]. It has been shown that both oxidative stress and p53 are activated in kidney cells by cisplatin treatment [14], and conversely, the inhibition of either oxidative stress or p53 significantly mitigates the cisplatin-induced cytotoxicity. These findings suggest that both oxidative stress and p53 function are prodeath signals, contributing to the nephrotoxicity of cisplatin. In their examination of the role of p53 activation in the regulation of cisplatin-mediated oxidative stress, Yuan et al. [14] demonstrated that knocking down p53 leads to a significant reduction of cisplatin-induced ROS generation and cell death induction in HK2 rental cells, thus suggesting that the activation of p53 promotes the oxidative stress and cell death induced by cisplatin. However, scavenging ROS attenuated cisplatin-induced p53 activation, thereby indicating that a positive feedback loop exists between the generation of ROS and the activation of p53 in response to cisplatin exposure. Further study is required to ascertain which of these is the primary event in cisplatin-induced nephrotoxicity.

Similarly, in the case of doxorubicin (DOX), a topoisomerase II inhibitor widely used in chemotherapy due to its efficacy in combating a wide range of cancers, the induction of cardiotoxicity is a major safety issue, with oxidative stress the most probable mechanism for its cardiotoxic effect [40]. Using wild-type and p53 homozygous knockout (p53(-/-)) mouse models, Velez et al. demonstrated that the oxidative stress in the mitochondria of cardiac tissue was induced only in the DOX-treated wild-type mice but not in the p53 knockout mice [39]. Accordingly, the mitochondrial injury of cardiac issues by DOX was significantly decreased in the absence of p53. These results clearly suggest that p53 exerts prooxidant activity and plays a critical role in DOX-induced cardiotoxicity. The prooxidant activity of p53 also has been reported to contribute to triptolide-induced cardiotoxicity in vitro and in vivo, while the p53 antagonist pifithrin-*α* could ameliorate triptolide-induced apoptosis by suppressing ROS accumulation in primary cardiomyocytes H9c2 cells [41].

Colistin, also known as polymyxin E, could be the first choice in the treatment of infections caused by multidrug-resistant Gram-negative bacteria [42]; however, its use is limited by nephrotoxicity and neurotoxicity. Lu et al. [43, 44] demonstrated that colistin treatment induced cell autophagy and apoptosis via a significantly increased p53 expression level and the accumulation of ROS in PC-12 cells. Moreover, the JNK activator anisomycin enhanced the levels of p53 and ROS above those of colistin alone. However, the silencing of p53 by siRNA before colistin and anisomycin treatment substantially reduced ROS production, thereby demonstrating the prooxidant activity of p53 [43, 44].

In addition to the side effects of therapeutic drugs, p53-mediated oxidative stress has also been noted in toxicant-induced toxicities. Patulin, a mycotoxin produced mainly by Aspergillus and Penicillium, is commonly found in moldy fruits and their derivative products [45], and it has been suggested that the induction of oxidative stress could play an important role in patulin-induced diverse toxic effects [46]. Previous research has shown that the inhibition of p53 by RNAi results in significantly ameliorated ROS generation, accompanied by a decrease in the extent of DNA damage and apoptosis induced by patulin in human embryonic kidney (HEK) 293 cells, thus indicating the contribution of p53 to patulin-induced oxidative stress [12]. Moreover, these in vitro findings were further validated in a subsequent animal study in which it was revealed that the oxidative stress induced by patulin in the kidney tissues was significantly attenuated in p53-knockout mice compared with that found in p53-WT mice.

Glycerol is a simple polyol compound often used in the food, medical, and pharmaceutical industries and in personal care preparations [47]. However, this agent is toxic at high concentrations, and glycerol-induced injury is commonly used as an experimental model of muscle adipogenesis or acute kidney injury [48]. Oxidative markers and the expression of phosphorylated-p53 were found to be increased in the kidneys of glycerol-treated rats [49], while the inhibition of p53 with pifithrin-*α* (PFT*α*) significantly reduced the expression of oxidative markers and glycerol-induced renal tubular injury in rats. These findings indicate that p53-mediated oxidative stress can contribute to glycerol-induced kidney injury.

Silibinin is a major active constituent of silymarin, and many studies have reported on the involvement of ROS and p53 in silibinin-activated pathways [50–52]. In HeLa cells, silibinin was found to induce ROS generation and activate p53. However, while the silibinin-induced ROS generation could be reduced by PFT*α* treatment, silibinin could not induce ROS generation in the p53-mutated human epithelial tumor A431 cell. These findings further verify the crucial role of p53 in silibinin-induced ROS generation [50].

## 3. Antioxidant Activity of p53 in Chemically Induced Oxidative Stress

In contrast to the prooxidant characteristics of p53, its antioxidant activity has also been reported to occur in chemically induced oxidative stress, as summarized in Table 2.

Acetaminophen (APAP) is one of the most commonly used analgesic drugs and is usually safe at the appropriate therapeutic doses. However, excessive doses can induce hepatotoxicity, and APAP overdose has become the leading cause of liver injury in the United States and most European countries [53, 54]. Huo et al. [36, 37] recently found that the inhibition of p53 by three different approaches in mice, namely, PFT*α*, knockdown of p53 expression with antisense oligonucleotide, and p53 knockout, leads to more severe liver injury by APAP. Conversely, the activation of p53 by its activator, nutlin-3a, resulted in ameliorated APAP-induced hepatotoxicity. These results clearly suggest that p53 plays a novel protective role in APAP-induced liver injury. Moreover, it was revealed that the protective effect of p53 on APAP-induced hepatotoxicity is attributed to its ability to inhibit the activation of JNK, a key mediator in APAP-induced oxidative stress [36, 37], (Huo et al. 2016).

1-Methyl-4-phenylpyridinium (ion) (MPP+) is a dopamine neurotoxin, which can induce parkinsonism [55]. MPP+ is widely used in the study of Parkinson's disease in various animal cell models [56, 57]. In SH-SY5Y cells, the expression of sestrin2, p53, and levels of ROS were induced by MPP+, while the increased expression of sestrin2 induced by MPP+ was abolished by the downregulation of p53 at both the mRNA and protein levels. Moreover, the knockdown of sestrin2 using siRNA was found to promote an increase in the levels of ROS induced by MPP+. These data indirectly demonstrate the antioxidant role that p53 plays in MPP+-treated SH-SY5Y cells [57].

The antioxidant function of p53 has also been found in the oxidative stress-related pathology of cardiovascular diseases. Oxidative stress induced by free fatty acids (FFA), for example, is considered to be a contributing factor to the metabolic syndrome-associated development of cardiovascular diseases. Exposure of FFA palmitate to human endothelial cells (ECs) caused the transcriptional inactivation of p53 via the inhibition of its acetylation, which, in turn, led to increased ROS generation and endothelial damage [58]. These in vitro findings were further validated in the in vivo model. Moreover, p53 has also been reported to provide some protection against palmitic acid-induced ROS accumulation and apoptosis in the HCT116 cell line [59].

Glucose, which is a simple sugar that provides the body with its primary source of energy, has been found to lead to p53 inactivation and ROS accumulation in human umbilical vein endothelial cells (HUVEC) and human aortic endothelial cells (HAEC). Wu et al. [60] concluded from these findings that high glucose-induced p53 phosphorylation at Thr55 contributes to the impairment of the endothelial antioxidant system.

p53 is also involved in oxidative stress induction caused by nitric oxide in vascular smooth muscle cells (VSMC). Popowich et al. [61] reported that exposure to nitric oxide induces a stronger apoptotic effect in p53−/− VSMC than that found in p53(+/+) VSMC, thus suggesting that p53 protects VSMC from nitric oxide-induced apoptosis. It was further demonstrated that the prosurvival activity of p53 in response to nitric oxide exposure is due to its ability to reduce ROS generation and cellular oxidative stress. These findings indicate that p53 can exert antioxidant activity, not only in its basal condition but also in activated conditions, which, in turn, contributes to its prosurvival functions.

## 4. Mechanisms of p53 Regulation of Chemically Induced Oxidative Stress

**Figure 1:**
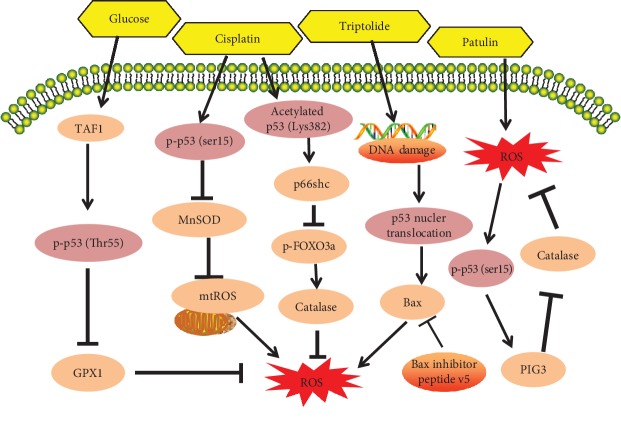
The prooxidant role of p53 signaling pathways in chemically induced oxidative stress (compiled from different cell types/lines and tissues): p53 activation in response to chemicals can increase intracellular oxidative stress and mitochondrial ROS levels. Chemicals cause p53-mediated prooxidant effects through mechanisms involved in the inactivation of GPX1, MnSOD, and FOXO3a and the activation of p66shc, PIG3, and Bax signals.

**Figure 2:**
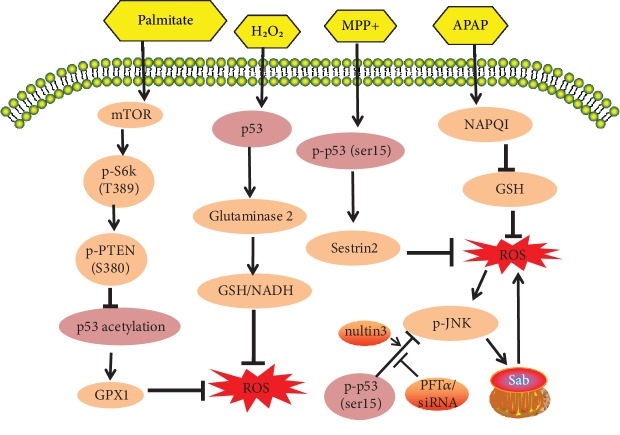
The antioxidant role of p53 signaling pathways in chemically induced oxidative stress (compiled from different cell types/lines and tissues): p53 activation can suppress the harmful effects of chemically induced intracellular ROS generation and oxidative stress. Chemicals cause p53-mediated antioxidant effects through a variety of mechanisms, including the activation of glutaminase 2 and GPX1, upregulation of sestrin2, and dephosphylation of JNK.

Recent research has identified the activities of p53 in the regulation of ROS, along with the upstream and downstream regulators responsible for p53 antioxidant and prooxidant functions. Numerous studies have shown, for example, that p53 can exert prooxidant activity to promote oxidative damage through the regulation of its transcriptional targets, such as p53-inducible genes (PIGs), NCF2/p67phox, a cytosolic subunit of the NADPH oxidase enzyme complex [12, 62], p66shc [14, 63], and Bax [41] (Figure 1). In contrast, however, several other studies argue that p53 can function as an antioxidant factor to inhibit oxidative stress through the regulation of several redox-related proteins, such as MnSOD [64], GPX1 [58, 64], Sestrins [57, 65], JNK [36, 37], glutaminase 2 [66] (Figure 2), and TIGAR [67].

### 4.1. p53 Exerts Prooxidant Activity through the Regulation of p66shc

P66shc, a splice variant of p52shc/p46shc, is a cytosolic adapter protein that transduces mitogenic signals from activated receptors to Ras [68]. p66shc has been reported as a novel biomarker of tubular oxidative injury in patients with diabetic nephropathy [68]. It has been well documented that the p53-p66shc signaling pathway plays a critical role in regulating the steady-state levels of intracellular oxidants and oxidative damage [69, 70]. Moreover, p53 activation induces the upregulation of p66shc protein by increasing its stability [71].

The role of p66shc in chemically induced p53-mediated oxidative stress was examined in a study by Yuan et al. [14] in which it was shown that p53 is activated by cisplatin treatment accompanied by increased p66shc expression and ROS generation in renal cells. The inhibition of p53 by RNAi significantly attenuates the cisplatin-induced upregulation of p66shc, whereas the knockdown of p66shc decreases the level of ROS. These findings suggest that the p53-mediated upregulation of p66shc contributes to cisplatin-induced oxidative stress in kidney cells. The authors also demonstrated that the prooxidant activity of p66shc is associated with the inhibitory phosphorylation of FOXO3a, a transcriptional factor that regulates antioxidant enzymes, MnSOD and catalase. Furthermore, they revealed that p53 inactivation by its inhibitor PFT*α* downregulates p66shc expression in vivo, a finding which was consistent with the results obtained in the cell culture model.

### 4.2. p53 Exerts Prooxidant Activity by Targeting PIG3

In 1997, Polyak et al. [72] used the serial analysis of gene expression to identify a series of p53-inducible genes (PIG genes) involved in ROS generation. Their findings established the first clear connection between p53 and ROS generation. p53-inducible gene 3 (PIG3 or TP53I3) is such one gene, which is a relative of NADPH-quinone oxidoreductase, a potent ROS generator [73]. PIG3 has been found to physically bind to and inhibit the activity of catalase, which, in turn, contributes to the prooxidant activity of PIG3 [74, 75].

It has been shown that mycotoxin patulin induces p53-mediated oxidative stress in kidney cells [12]. To investigate the downstream mediator of p53-dependent oxidative stress in this study, we analyzed the role of PIG3 and found, first, that it is increased through a p53-dependent manner and, in addition, that silencing PIG3 through RNAi results in significantly decreased patulin-induced ROS generation, which is associated with the upregulation of catalase. These in vitro findings were further validated in the p53 wild-type/knockout mouse model. PIG3 was not detectable in p53 KO mice; however, it was upregulated by patulin treatment in the kidney samples of p53 wild-type mice.

### 4.3. p53 Regulates Oxidative Stress by Targeting GPX1

The GPXs are a group of selenocysteine-containing antioxidant enzymes [74], of which five different isoforms have been identified in mammals. Among them, GPX1, a cytosolic form of GPX, is the major isoform that catalyzes the decomposition of hydrogen peroxide to molecular oxygen and water [76]. It has been shown that GPX can be transcriptionally regulated by p53 [64], and it is, therefore, reasonable to conclude that the chemicals that can inhibit p53 may cause oxidative stress via the suppression of the p53-GPX pathway. In the study of endothelial cells, for example, oxidative stress was induced by high levels of glucose, resulting in endothelial dysfunction and tissue damage [60]. Exposure to high levels of glucose increased the phosphorylation of p53 at Thr55 through TAF1 kinase. The phosphorylated p53 then dissociated from the GPX1 promoter, leading to the transcriptional reduction of GPX1. The inactivation of TAF1 or inhibition of p53 Thr55 phosphorylation, thus, suppresses high glucose-induced ROS generation accompanied by increased GPX1 expression.

### 4.4. p53 Exerts Antioxidant Activity by Inhibiting c-Jun N-Terminal Kinase (JNK)

The mitogen-activated protein kinase (MAPK) cascades are evolutionary conserved intracellular signal transduction pathways. They are involved in the transmission of mitogenic signals from the cell surface to regulatory targets and also regulate multiple cellular processes, including proliferation, differentiation, and cell death [77–79]. To date, three key mammalian MAPK cascades have been identified, namely, extracellular signal-regulated kinase 1 and 2 (ERK1/2), c-Jun N-terminal kinase (JNK), and p38 [80]. Each of these consists of three enzymes, MAPK, MAPK kinase (MAPKK), and MAPK kinase kinase (MAPKKK), which are all sequentially activated through phosphorylation. The activation of MAPKs, especially the stress-activated kinase, JNK, is common under conditions of oxidative stress. In most cases, MAPK activation occurs downstream of ROS generation [81, 82]; however, activated MAPKs can also positively regulate ROS generation in certain conditions [44, 83], (Huo et al. 2016).

It is well established in APAP-induced liver injury that APAP induces the rapid production of the reactive metabolite NAPQI, followed by the depletion of glutathione (GSH) and generation of ROS, consequently resulting in the activation of JNK [83, 84]. The activated JNK then translocates to the mitochondria and interacts with the mitochondrial outer membrane protein Sab, a scaffold protein, which leads, in turn, to the disruption of the mitochondrial electron transport chain and persistent ROS generation and JNK activation through a self-sustaining MLK3/ASK1⟶p-MKK4⟶p-JNK⟶Sab⟶ROS pathway (Huo et al. 2016). The role of p53 in the regulation of this pathway was recently determined when JNK activation was found to be increased by the inhibition of p53 through either a genetic or pharmacological approach [54]. Conversely, the activation of JNK was decreased when p53 was induced by the activator nutlin-3a, an inhibitor of MDM2 which, in turn, inhibits p53 degradation. These findings, thus, suggest that p53 can suppress APAP-induced oxidative stress by targeting JNK.

### 4.5. p53 Functions as an Antioxidant via the Upregulation of Sestrin2

As a family of evolutionarily conserved proteins, Sestrins can suppress reactive oxygen species and provide cytoprotection against oxidative stress [85–87]. Three different Sestrin proteins have been found to exist in mammals, namely, sestrin1, sestrin2, and sestrin3, of which sestrin2 is known to play a crucial role in modulating the production of ROS.

Sestrins have been widely reported to link p53 with redox regulation [57, 65, 88]. Zhou et al. [57] found that 1-methyl-4-phenylpyridinium (MPP+) increased ROS production in SH-SY5Y while also increasing the expression of sestrin2 and phosphorylation of p53 at ser15. The upregulation of sestrin2 at both the mRNA and protein levels induced by MPP+ was, however, mitigated by the knockdown of p53. Moreover, silencing sestrin2 using small interference RNA significantly promoted MPP+-mediated ROS generation and neurotoxicity. These findings, therefore, suggest that p53 plays an antioxidant role through the modulation of sestrin2 expression.

### 4.6. p53 Enhances Antioxidant Defense through Upregulating Glutaminase 2

Glutaminase (GLS), which is the initial enzyme in glutamine metabolism, has two isoenzymes, namely, GLS1 and GLS2 [89]. GLS2 decreases ROS levels in cells through glutathione-dependent antioxidant defense, which, in turn, protects cells from oxidative stress [90].

GLS2 has also been reported to act as a mediator of p53's role in energy metabolism and antioxidant defense [66, 91]. Treatment with H_2_O_2_ induces GLS2 mRNA significantly in HCT116 p53+/+ cells but not in p53−/− cells, thus revealing that H_2_O_2_ induces GLS2 mRNA levels in a p53-dependent manner. It has also been found that p53 can regulate GLS2 basal expression levels in HepG2 cells. Moreover, H_2_O_2_ accumulates more ROS in HCT116 p53−/− cells than in p53+/+ cells, while silencing GLS2 can also increase ROS accumulation in HCT116, H1299, and HTB-15 cells. These findings demonstrate that p53 regulates cellular antioxidant defense through the modulation of GLS2 expression.

## 5. Targeting p53 for the Modulation of Chemically Induced Oxidative Stress and Toxicity

As has been described above, p53 has the ability to exert either prooxidant activity to mediate chemically induced apoptosis or antioxidant activity to protect cells from chemically induced cytotoxicity. Therefore, p53 is considered a reasonable target through which to suppress chemically induced oxidative stress and toxicity [36, 37, 43, 60]. p53 is activated by APAP treatment, for example, and functions as an antioxidant to inhibit APAP-induced oxidative stress and liver injury by inactivating the JNK-Sab-ROS loop. These findings provide the rationale for p53 as a target to treat APAP-induced hepatotoxicity. Indeed, nutlin-3a, a p53 activator, was demonstrated in previous work by the current study's authors to significantly ameliorate APAP-induced liver injury in a mouse model.

As mentioned above, p53 is an important transcription factor, and it transcriptionally regulates multiple downstream targets that are involved in regulating various biological processes such as cell cycle, apoptosis, and DNA repair. Targeting p53 could offer protection against chemical-induced oxidative stress and toxicity, but meanwhile, it might affect physiological functions of p53 to produce certain side effects. To avoid these unwanted influences, the manipulation of p53 needs to be optimized so that the basal p53 activity can be maintained to enable its physiological functions unaffected. For example, p53 was activated by PAT, which induced ROS generation and cytotoxicity [12]. Titration experiment can be performed to determine the concentration of p53 inhibitor that can effectively inhibit PAT-induced p53 activation but do not influence basal activity of p53 and its essential biological functions.

## 6. Concluding Remarks

Oxidative stress can lie either upstream or downstream of p53 activation, which has been found capable of exerting either prooxidant activity to mediate chemically induced apoptosis or antioxidant activity to protect cells from chemically induced cytotoxicity. The determinants of this paradoxical role of p53 in the regulation of oxidative stress remain elusive. It is generally believed that, under physiological conditions and the conditions of mild stress, p53 exerts antioxidant activity to inhibit ROS generation and protect cells from oxidative DNA damage, while under conditions of severe stress, activated p53 enhances oxidative stress and promotes cell death. Nonetheless, this speculation cannot account for all stress cases. p53 is activated in APP-treated liver cells, for example, and APAP treatment has been found to cause severe oxidative stress followed by severe liver injury. However, activated p53 is reported to play an antioxidant role in this stressed condition, which suggests the involvement of additional factors in determining the prooxidant or antioxidant function of p53.

Further investigations into the activity of p53 should address the following issues:

1. Evaluation of the influence of the source or type of ROS on the pro- or antioxidative activity of p53
2. Assessment of the role of levels of p53 activation in response to chemical exposure in its pro- or antioxidative function
3. Determination of whether the cell type is a contributing factor for the controversial function of p53 in regulating oxidative stress
4. Investigation of the novel mechanisms underlying p53-regulated oxidative stress in response to chemical exposure

A better understanding of the factors prompting the activity of p53 as either prooxidant or antioxidant will further the development of this important cell tumor antigen as a precisely utilized target to manage the oxidative stress-mediated toxic responses to chemical exposure.

## Figures and Tables

**Table 1:** The prooxidant activity of p53 in chemically induced oxidative stress.

Chemical	Cell lines/animals	Assays	Result	Ref.
Cisplatin	HK2 cells, 25 nM, 24 h. Mice, 20 mg/kg, i.p., 72 h.	mtROS, p53, MnSOD, p66shc	(i) p53 siRNA inhibited cisplatin-induced mtROS and cytotoxicity in HK2 cells. (ii) PFT*α* blocked cisplatin-induced oxidative stress and apoptosis in the kidney.	[60]
Doxorubicin	C57BL/6 mice wild type and p53−/−, 20 mg/kg, i.p., 3 d.	4HNE, p-JNK, Bcl2	(i) The absence of p53 significantly reduced oxidative damage in mitochondria and DOX-induced cardiac toxicity.	[39]
Triptolide	H9c2 cells, 160 nM, 24 h. C57BL/6 mice wild type, p53−/−, 1.2 mg/kg i.v. 24 h.	ROS, Bcl2 family	(i) PFT*α* pretreatment significant repression of ROS accumulation induced by TP in the H9c2 cell. (ii) p53 deficiency abolishes the cardiotoxicity induced by triptolide treatment.	[41]
Colistin	PC-12 cells, 125 *μ*g/mL colistin	ROS	(i) Silencing of p53 caused a tremendous decrease in the ROS levels in PC-12 cells with colistin plus anisomycin.	[43, 44]
Patulin	HEK293, MEF cells wild type and p53−/−, 7 *μ*M, 24 h. C57BL/6 mice wild type and p53−/−, 2.5 mg/kg, i.p., 1, 3, 6, and 12 h.	ROS, catalase activity, comet assay	(i) Inactivation of p53 decreased ROS generation in response to patulin exposure in vitro. (ii) p53 activation played a prooxidant role in patulin-induced oxidative stress.	[12]
Glycerol	Wistar rats, 50% glycerol (7 mL/kg), intramuscular injection, 24 h.	MnSOD, ROS, GPX1, HO-1, GSH NQO-1	(i) PFT*α* attenuates ROS formation, tubular injury, and renal functional deterioration.	[49]
Silibinin	HeLa cells, A431 cells (lacked functional p53), 50 *μ*M, 12 h.	ROS, p-JNK, MMP	(i) Silibinin could not induce ROS generation without normal functional p53.	[50]

**Table 2:** The antioxidant activity of p53 in chemically induced oxidative stress.

Chemical	Cell lines/animals	Assays	Result	Ref.
Acetaminophen	C57BL/6 mice wild type and p53−/−, APAP, 300 mg/kg, i.p., 1, 2, 4, and 24 h. PFT*α*, 2.2 mg/kg, i.p., nutilin-3a, 10 mg/kg, gavage p53 ASO, 50 mg/kg, i.p.	ROS, NAPQI adducts, Sab, p-JNK, ALT	(i) Sustained JNK activation leading to increased mitochondrial ROS. (ii) p53 inhibition enhanced sustained JNK activation, while the JNK was suppressed with the p53 activator.	[36, 37]
Palmitate	HUVEC and HAEC cells, 0.4 mM PA, 8–16 h. C57BL/6J mice, HFAD-fed, 12 w	ROS, NO, GPX1, aortic lesions	(i) Palmitate-siRNA rescued the inhibition of p53 binding to GPX1 promoter and then blocked PA-induced ROS formation. (ii) HFAD-induced oxidative stress and vascular damage via PTEN nuclear export, p53/GPX1 inhibition.	[58]
Glucose	HUVEC and HAEC cells, 20 mM, 0–48 h	ROS, TAF1	(i) TAF1-mediated p53 phosphorylation at Thr55 and GPX1 suppression plays a critical role in ROS accumulation.	[60]
Nitric oxide	VSMC wild type and p53−/−, DETA/NO, 1 mM, 24 h	ROS, SOD-2, PRx-3, and TRx-2	(i) p53−/− VSMC have increased levels of ROS at baseline and following exposure to NO compared with p53+/+ VSMC. (ii) p53 have antioxidant properties and antiapoptotic functions in VSMC.	[61]
1-Methyl-4-phenylpyridinium	SH-SY5Y, 100 *μ*M, 24–72 h	ROS, 4HNE	(i) Increased expression of sestrin2 induced by MPP+ was abolished by downregulation of p53. (ii) Inhibition of sestrin2 by siRNA significantly promoted increased levels of ROS induced by MPP+.	[57]

## Abbreviations

Nrf2: Nuclear factor E2-related factor
GSH: Glutathione
NADH: Nicotinamide adenine dinucleotide hydrate
MnSOD: Manganese superoxide dismutase
GPx1: Glutathione peroxidase 1
TIGAR: TP53-inducible glycolysis and apoptosis regulator
PIGs: p53-induced genes
P66shc: 66 kD isoform of the shc (Src homology and collagen)
ROS: Reactive oxygen species
MAPK: Mitogen-activated protein kinase
TAF1: TBP-associated factor 1
FOXO3a: Forkhead box O3
NAPQI: N-Acetyl-p-benzoquinone imine.
